# Noxious Stimulation Induces Acute Hemorrhage and Impairs Long-Term Recovery after Spinal Cord Injury (SCI) in Female Rats: Evidence Estrous Cycle May Have a Modulatory Effect

**DOI:** 10.1089/neur.2021.0055

**Published:** 2022-01-31

**Authors:** Rachel E. Baine, David T. Johnston, Misty M. Strain, Melissa K. Henwood, Jacob A. Davis, Joshua A. Reynolds, Erin D. Giles, James W. Grau

**Affiliations:** ^1^ Department of Psychological and Brain Sciences, Texas A&M University, College Station, Texas, USA.; ^2^ Department of Cellular and Integrative Physiology, University of Texas Health Science, San Antonio, Texas, USA.; ^3^ Department of Neuroscience, Cell Biology, Anatomy, University of Texas Medical Branch, Galveston, Texas, USA.; ^4^ Department of Nutrition, Texas A&M University, College Station, Texas, USA.

**Keywords:** females, hemorrhage, pain, polytrauma, spinal cord injury

## Abstract

Spinal cord injuries (SCIs) are often the result of traumatic accidents, which also produce multiple other injuries (polytrauma). Nociceptive input from associated injuries has been shown to significantly impair recovery post-SCI. Historically, work in our laboratory has focused exclusively on male animals; however, increasing incidence of SCI in females requires research to determine whether pain (nociceptive) input poses the same risk to their recovery. Some animal studies have shown that females demonstrate greater tissue preservation and better locomotor recovery post-SCI. Given this, we examined the effect of sex on SCI recovery in two pain models—intermittent electrical stimulation (shock) to the tail or capsaicin injection to the hindpaw. Female rats received a lower thoracic contusion injury and were exposed to noxious stimulation the next day. The acute effect of noxious input on cardiovascular function, locomotor performance, and hemorrhage were assessed. Treatment with capsaicin or noxious electrical stimulation disrupted locomotor performance, increased blood pressure, and disrupted stepping. Additional experiments examined the long-term consequences of noxious input, demonstrating that both noxious electrical stimulation and capsaicin impair long-term recovery in female rats. Interestingly, injury had a greater effect on behavioral performance when progesterone and estrogen were low (metestrus). Conversely, nociceptive input led to a greater disruption in locomotor performance and produced a greater rise in blood pressure in animals injured during estrus.

## Introduction

Injury to the spinal cord can leave individuals with long-term debilitating sensory and motor deficits. Spinal cord injuries (SCIs) result from a variety of causes, including vehicular accidents, falls, or violence, which often lead to additional tissue damage (polytrauma) that will drive pain (nociceptive) fibers after injury. We have examined the effect of noxious stimulation using an animal model (rats) and a contusion injury to the lower thoracic spinal cord. Noxious stimulation 1–4 days after a moderate injury impairs long-term recovery, fosters the development of chronic pain, and increases tissue loss. Stimulation appears to have a damaging effect, in part, because it fuels hemorrhage at the site of injury.^1–4^ By identifying the mechanisms behind these pathways, the deficits they elicit may be prevented.

This line of inquiry has been pursued using two different pain models—uncontrollable electrical stimulation (shock) to the tail and intradermal capsaicin injection to the hindpaw.^2,5,6^Activation of nociceptive fibers by these noxious stimuli initiates a cascade of events that ultimately compound the effect of the initial mechanical damage done to the spinal cord (primary injury).^7^ In the hours to days after the primary injury, the neurobiological processes contributing to the secondary injury unfold, leading to expansion of the lesion site, fragmentation of capillaries resulting in hemorrhage, and deficits in behavioral function.^6,8,9^ Pain has also been shown to produce an elevation of blood pressure/flow that may fuel the expansion of hemorrhage at the site of injury.^10,11^

Historically, our laboratory has conducted this research using male animals, because of the prevalence of SCI sustained by male patients. Other laboratories have often used female animals, given their comparative resistance to physiological complications like urinary tract infections. However, recent work suggests that our conclusions may hinge on whether male or female animals were tested. Indeed, studies of SCI in female animals have revealed a form of sex-dependent protection from injury-related processes, with increased tissue sparing and improved locomotor function relative to male controls.^12–14^ It has been suggested that female gonadal hormones, particularly estrogen, may play a role in protecting females against the negative effects of secondary injury. For example, when treated with estradiol, male rats demonstrated improved behavioral outcomes and less cytokine expression compared to vehicle-treated animals.^15–18^

Given the effects of shock and capsaicin previously observed in male animals, the current study was conducted to determine whether the same deficits are observed in female rats. We evaluated the effects of these treatments 24 h after female animals received a moderate contusion of the lower thoracic (T11–T12) spinal cord. Additionally, we went beyond past work to examine the role of blood pressure post-SCI and nociceptive input. Disruption of the cardiovascular system after SCI has extensive implications for recovery post-injury, through dysregulated hemodynamics and exacerbation of secondary injury.^19–22^ Our hypothesis was that a rise in blood pressure and/or flow is related to an increase in hemorrhage, which would increase tissue loss at the site of injury and undermine long-term recovery.

We also assessed the role of estrous cycle in recovery from SCI and its relationship in response to nociceptive stimulation. Injury to the spinal cord has been shown to affect the reproductive cycle in female animals; after SCI, the estrous cycle is disrupted, with animals either arrested in a single stage or abnormally cycling through all four stages (proestrus, estrus, metestrus, and diestrus).^23–25^ This effect is attributed to the severity of the injury itself and has been positively correlated with the percentage of damage to the ventromedial white matter.^23^ Post-SCI, gonadal hormones have been shown to modulate pain through complex mechanisms, enhancing reactivity in some cases while reducing it in others.^26–30^ This bidirectional effect on pain response has been linked to fluctuations in circulating sex hormones and, importantly, may predict when the system is particularly vulnerable to injury and the effects of noxious stimuli.

The influence of varying gonadal hormones in female animals has been explored in two ways. The most common approach involves administering estradiol or progesterone manually to gonadectomized animals, isolating the effect of a particular hormone over a range of concentrations.^31^ An alternative method takes advantage of the natural variation in gonadal hormones across the estrous cycle. In this case, the estrous cycle is tracked in intact female animals and treatment is applied across animals at different points in the estrous cycle. Whereas administering sex hormones pharmacologically allows for greater manipulation of plasma levels, the second approach enables observation of the natural fluctuations of hormones in an intact animal. We used the latter strategy in experiments 2–4 to examine whether estrous cycle affects the behavioral/physiological consequences of injury and noxious stimulation. If sex hormones have a protective effect, injury may have a greater effect when given at a point when levels are low (metestrus). Finally, the tracking cycle before injury allowed us to evaluate whether the normal cycle was disrupted by injury and/or exposure to pain (experiments 3 and 4).

## Methods

### Subjects

Adult (12-week) female Sprague Dawley rats were obtained from Envigo (Houston, TX). Weights at the start of treatment ranged from 218 to 270 g (mean = 244). Animals were housed in a vivarium with a 12-h light-dark cycle and food and water provided *ad libitum*. Experiments were carried out according to standards set by the National Institutes of Health (NIH) for laboratory animal care and use, and experiments were approved by the University Laboratory Animal Care Committee at Texas A&M University. Every effort was made to minimize pain and suffering experienced by the animals.

### Contusion surgery

Animals were given a moderate contusion to the spinal cord at T11–T12 using the NYU Multicenter Animal Spinal Cord Injury Study (MASCIS) device. Before surgery, animals were anesthetized with 5% isoflurane and medical oxygen, then maintained at 2–3% isoflurane during surgery. A laminectomy was performed at the T12 vertebra to expose the spinal cord. The MASCIS device was used to secure and center the animal's spinal cord before dropping a 10-g weight from a 12.5-mm height to contuse the spinal cord. Animals were administered penicillin (100,000 U/kg) to prevent infection and 3 mL of saline to compensate for fluid loss during surgery. After surgery, animals recovered overnight in a temperature-controlled room (25°C), with food and water *ad libitum*. After experimentation, animals were euthanized with a lethal injection of pentobarbital (100 mg/kg, intraperitoneal [i.p.]).

### Health checks

During the 28-day post-treatment recovery period in experiments 3 and 4, animals were monitored daily for signs of excessive weight loss, dehydration, infection, and autophagia. If weight loss exceeded 25% of preoperative weight, animals were euthanized with pentobarbital (*n* = 5). Dehydration was treated with a 1-mL injection of saline (intraperitoneal; i.p.). In cases of severe autophagia, antibacterial ointment (Neosporin) was applied, followed by a liquid bandage (New-Skin) and taste-deterrent spray (Grannicks Bitter Apple). Penicillin was administered daily (100,000 U/kg, i.p.) upon display of autophagia or urinary tract infection. Incidence of autophagy over the 28-day recovery period was comparable across the pain (7 of 17) and no pain (6 of 17) conditions. Duration of autophagy was unrelated to the change in locomotor performance (*r* = 0.098, *p* > 0.05). Throughout the recovery period, animals' bladders were manually voided twice-daily until voluntary control was re-established (six consecutive expressions with no urine).

### Estrous cycle analysis

For each experiment, estrous cycle was monitored to balance day of injury by stage of cycle. Cell samples were collected daily at 10:00 am for 10 days before injury, using a sterile cotton swab moistened with deionized water. Smears were then analyzed by light microscopy at 10 × magnification and identified as either proestrus, estrus, metestrus, or diestrus.^32–35^ During experiments 3 and 4, samples were collected daily throughout the animals' recovery.

### Locomotor function

Before surgery, animals were acclimated to a 45-inch plastic pool on 3 separate days for 4 min. Locomotor function was then assessed by researchers blinded to treatment condition, beginning the day after contusion using the Basso, Beattie, Bresnahan (BBB) scoring system.^36^ To assure that an adequate sample of locomotor performance was obtained, particularly after noxious stimulation when animals are less active, each animal was observed for 4 min. For acute studies, animals were scored before and at time points immediately after treatment. For experiments 3 and 4, animals were assessed each day for the first 7 days, on the 10th day, and then once a week until day 28 post-treatment. BBB scores were converted as described by Ferguson and colleagues.^37^ This transformation improves the metric properties of the scale enabling the application of parametric analyses. Key milestones on this scale include: 1) slight movement of one or two joints of the hindlimb; 3) slight movement of two joints and extensive movement of a third; 5) extensive movement of all three joints; 7) weight support; 9) frequent weight-supported plantar stepping; and 12) plantar stepping with consistent forelimb/hindlimb coordination.

### Blood pressure analysis

Before surgery, rats were acclimated on 3 separate days to the blood pressure assessment system. Animals were placed in a clear acrylic tube with an adjustable nose cone atop a warming platform (Kent Scientific Corporation, Torrington, CT). Blood pressure measurements were obtained using the CODA High Throughput Noninvasive Blood Pressure system and data acquisition software (Kent Scientific). This system yields six measures of cardiovascular function: systolic blood pressure; diastolic blood pressure; mean arterial blood pressure; heart rate; blood flow; and blood volume. Before analyses,^11^ we have shown that the three measures of blood pressure are highly correlated (all *r*s > 0.93), and for this reason, we typically present just one (systolic). Likewise, there is a high correlation between flow and volume (*r* = 0.98), and, for this reason, just one measure (flow) is presented here. Because hemorrhage was related to blood flow and systolic blood pressure, but not heart rate,^11^ heart rate data are not presented here.

### Nociceptive input

#### Intermittent tail shock

Animals were restrained in opaque Plexiglass tubes in a soundproof box. Tails were secured to an electrode placed ∼4 cm from the tip of the tail. Electrode gel was used to ensure contact between the electrode and tail. For 6 min, electrical stimulation was given in a variable-spaced pattern (100-ms pulse, intertrial interval 0.2–3.8 sec, and 60-Hz alternating current).^38^ Controls were similarly restrained, but received no shock.

#### Capsaicin injection

Capsaicin (3%) was dissolved in 5% Tween-20, 5% ethanol, and 90% saline (0.09% NaCl). Before administration, the solution was slightly heated and vortexed to ensure that the capsaicin was completely dissolved. The vehicle contained all the same components except capsaicin. For treatments, 0.05 mL of drug or vehicle was injected intradermally with a 27-gauge needle into the dorsal surface of the animal's hindpaw.^5^ Animals were restrained in opaque Plexiglass tubes for the injection and for 6 min after, to maintain consistency between experimental treatment groups.

### Protein analysis

#### Tissue collection

After a lethal dose of pentobarbital (100 mg/kg), a 1-cm section of spinal cord centered on the injury site was collected. Samples were then flash-frozen in liquid nitrogen and stored at −80°C until processed. Protein was extracted using radioimmunoprecipitation assay lysis and extraction buffer, according to the manufacturer's instructions.

#### Spectrophotometric analysis

Spectral analyses for free hemoglobin were conducted from protein extracts from lesion tissue. Spectrophotometric absorbance was measured from 1.5 μL of protein extract (NanoDrop;; ThermoFisherScientific, Waltham, MA) at an absorbance of 420 nm to measure hemoglobin content.^7,39^

#### Immunoblotting

After protein extraction, a Bradford assay quantified the concentration of protein per sample. Samples were then diluted with 4 × Laemeli buffer to a final concentration of 3 μg/μL. Western blots were run on pre-cast 26-well Criterion gels (Bio-Rad, Hercules, CA). Diluted protein samples were heated to 96°C for 10 min before 10 μL of each sample was loaded into the wells. Electrophoresis was then performed at 180 V for 75 min. Proteins were then transferred to a polyvinylidene difluoride membrane for 1 h at 100 V. Membranes were then blocked in milk for 1 h before incubating overnight in primary antibody (α-hemoglobin; 1:1000; ab92492; RRID, AB10561594; Abcam, Cambridge, MA) at 4°C. The next day, membranes were washed three times with Tris-buffered saline (TBS) with Tween-20, then once more in TBS, before incubation for 1 h in secondary antibodies (goat antirabbit; 1:5000; ab 258649; RRID, AB228341; Sigma-Aldrich, St. Louis, MO) at room temperature. Finally, blots were imaged using enhanced chemiluminescence.

### Experimental designs

#### Experiment 1: acute effects of intradermal capsaicin injection in female rats

Twenty-four hours after moderate contusion injury at T12, animals were assessed for baseline locomotor function and blood pressure values. Animals then received either 0.05 mL of capsaicin or its vehicle by intradermal injection to the dorsal surface of the hindpaw and were restrained for 6 min. Immediately after, and at 1, 2, and 3 h after treatment, locomotor function and blood pressure were measured. After animals were euthanized with a lethal dose of pentobarbital, the spinal cord was collected and flash-frozen for subsequent protein analysis using spectrophotometry and immunoblotting.

#### Experiment 2: acute effects of cutaneous electrical stimulation in female rats

Similar to experiment 1, animals received moderate contusion injuries at T12 and were treated 24 h later. Baseline locomotor function and blood pressure analysis was also performed before treatment. Animals then received either 6 min of intermittent electrical stimulation to the tail or no stimulation. Locomotor function and blood pressure were assessed immediately after treatment and at hours 1, 2, and 3. Animals were then euthanized with a lethal dose of pentobarbital. Tissue from the spinal cord was collected and protein extracted for analysis with spectrophotometry and immunoblotting.

#### Experiment 3: long-term effects of intradermal capsaicin injection in female rats

Animals were treated according to the procedures described in experiment 1. Twenty-four hours after moderate contusion injury at T12, animals were assessed for locomotor function, then injected with capsaicin or its vehicle to the hindpaw. For 28 days after treatment, animals recovered and were assessed for locomotor recovery. BBB locomotor scores were recorded on days 1–7, 10, 14, 21, and 28. Weights and other health measures were recorded daily. Additionally, estrous cycle was monitored throughout the recovery period. On day 28, animals were then euthanized and perfused, and their spinal cords were collected for histological analysis.

#### Experiment 4: long-term effects of cutaneous electrical stimulation in female rats

Animals received intermittent electrical stimulation to the tail, as described in experiment 2. Similar to procedures described in experiment 3, rats recovered for 28 days and were assessed for locomotor recovery using the BBB locomotor scale on days 1–7, 10, 14, 21, and 28. Throughout recovery, weights and estrous cycle were monitored daily. On the last day, animals were euthanized and perfused and their spinal cords collected for histological analysis.

### Statistical anlysis

Data were analyzed using analysis of variance (ANOVA) or analysis of covariance (ANCOVA). A criterion of *p* < 0.05 was established as the threshold of statistical significance in all cases. Analyses were conducted using the jamovi statistical package, with medmod and jAMM modules installed.

## Results

### Nociceptive input negatively impacts acute locomotor function in female rats

Previous work has shown that the detrimental effects of nociceptive input—both noxious electrical stimulation and intradermal capsaicin—on locomotor function after SCI in male rats are evident within hours of treatment.^2,4,5^ We first assessed whether these acute effects are evident in female rats.

**FIG. 1.**
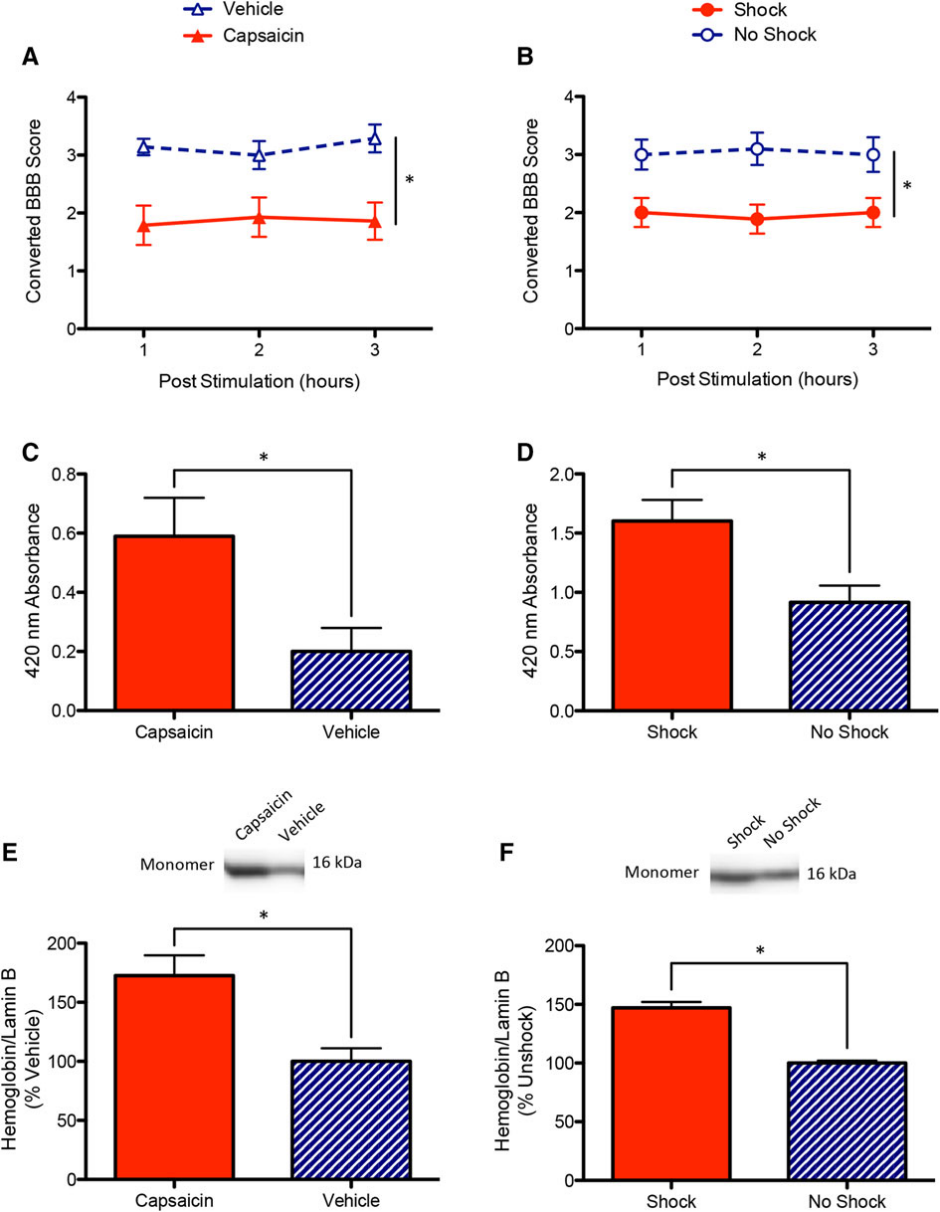
Acute effects of nociceptive input on locomotor function and hemorrhage in female rats. (**A**) Animals that received intradermal injection of capsaicin to the hindpaw showed an acute disruption in locomotor performance, relative to the vehicle controls, from 1 to 3 h after treatment (Post Stimulation). Symbols represent data from individual subjects. (**B**) Exposure to noxious electrical stimulation (Shocked) also produced a disruption in locomotor performance. (**C**) Spectrophotometric analysis found greater absorbance of protein samples at the wavelength corresponding to the absorbance of hemoglobin (420 nm) for animals treated with capsaicin. (**D**) Animals that were shocked also exhibited greater absorbance. (**E**) Western blot analysis found greater hemoglobin content at the site of injury in capsaicin-treated animals. (**F**) Western blot analysis also revealed greater hemoglobin in shocked animals. Representative immunoblots displaying greater labeling at 16 kDa (hemoglobin monomer) are provided above. *Indicates statistical significance (*p* < 0.05). Error bars represent standard error of the mean (Capsaicin, *n* = 7; Vehicle, *n* = 7; Shock, *n* = 8; No Shock, *n* = 8). BBB, Basso, Beattie, Bresnahan.

Experiment 1 found that an intradermal capsaicin injection in female rats after SCI produced an acute disruption in locomotor performance (Fig. 1A). Before treatment, baseline BBB scores did not differ (*F*_(1, 12)_ < 1.215, *p* > 0.05). After treatment, an ANCOVA, with baseline BBB score serving as the covariate, found that locomotor function was impaired in animals that received capsaicin injection (*F*_(1, 11)_ = 24.613, *p* = 0.0004).

Experiment 2 showed that exposure to noxious electrical stimulation (shock) also produced an acute disruption in locomotor function in female rats (Fig. 1B). Baseline BBB scores did not differ across groups (*F*_(1, 14)_ < 1.0, *p* > 0.05). After shock treatment, female rats exhibited poor locomotor performance relative to controls (*F*_(1, 13)_ = 12.448, *p* = 0.004).

### Both capsaicin and shock induce hemorrhage at injury site in female rats

After the 3 h of behavioral assessments in experiments 1 and 2, animals were euthanized and their spinal cord tissue collected for protein extraction and analysis. Past work has demonstrated increased amounts of hemorrhage at the injury site in male animals exposed to nociceptive stimuli.^6,40^ To determine the effects of capsaicin and shock on hemorrhage in female animals, the concentration of α-hemoglobin in samples was assessed with spectrophotometry and western blotting.

An ANOVA found that protein extracts from animals injected with capsaicin in experiment 1 exhibited greater absorbance at 420 nm, the wavelength associated with hemoglobin (*F*_(1, 12)_ = 6.77, *p* < 0.05; Fig. 1C). Immunoblotting was then used to confirm this effect. Levels of α-hemoglobin were significantly elevated in animals that received nociceptive input (*F*_(1, 12)_ = 10.873, *p* < 0.01; Fig. 1E).

Similarly, protein extracts from animals that received shock in experiment 2 exhibited greater absorbance at 420 nm (*F*_(1, 14)_ = 7.020, *p* < 0.05; Fig. 1D). Western blot analysis of α-hemoglobin content revealed increased levels in shocked animals (*F*_(1, 14)_ = 7.720, *p* < 0.05; Fig. 1F).

### Shock, but not capsaicin, elevates blood pressure in female rats

Past work suggests that nociceptive input after injury may expand the region of tissue loss after SCI in males because it induces a rise in blood pressure/flow that promotes hemorrhage at the site of injury.^10,11^ To examine whether a similar effect is observed in female rats, cardiovascular function was monitored for 3 h after exposure to capsaicin (experiment 1) or noxious electrical stimulation (experiment 2). Earlier analyses implicated systolic blood pressure and blood flow^11^; we focused on these measures.

**FIG. 2.**
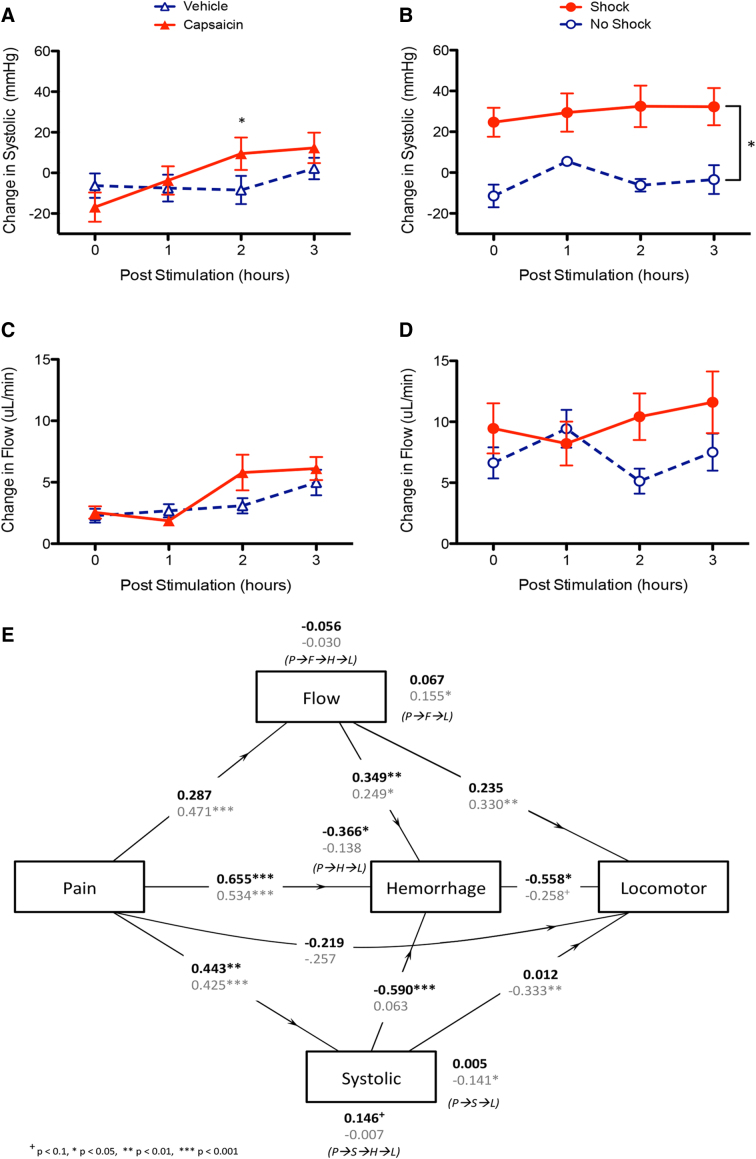
Acute effects of nociceptive input on measures of blood pressure in female rats. (**A**) Capsaicin treatment produced a modest increase in systolic blood pressure that was significant at 2 h. (**B**) Shocked animals had elevated systolic blood pressure relative to untreated (No Shock) controls. (**C**) Blood flow was not altered by capsaicin treatment. (**D**) Effect of shock treatment on blood flow approached statistical significance (*p* = 0.62). (**E**) Mediation analysis of nociception, blood flow, and systolic blood pressure on hemorrhage and locomotor function. The figure provides the standardized beta values, which vary between −1 and 1. These coefficients can be interpreted in a manner analogous to a correlation coefficient. A value close to zero suggests no relation; as the strength of the relation increases, the absolute value of the coefficient approaches 1. *Indicates statistical significance (*p* < 0.05). Error bars represent standard error of the mean (Capsaicin, *n* = 7; Vehicle, *n* = 7; Shock, *n* = 8; No Shock, *n* = 8).

Capsaicin treatment had a limited effect on blood pressure. Before injection of capsaicin in experiment 1, systolic blood pressure and flow values did not differ between groups (*F*s < 4.747, *p* > 0.05). An ANOVA analyzing changes in systolic blood pressure from baseline values found that there was an effect of capsaicin treatment that emerged over time, with the effect evident at 2 h after injections (*F*_(3, 36)_ = 3.737, *p* = 0.0195; Fig. 2A). There was no effect of capsaicin on blood flow after injection (*F*s < 1.906, *p* > 0.05; Fig. 2C).

As observed in male animals,^11^ exposure to noxious electrical stimulation (shock) had a more robust effect on cardiovascular function. By chance, animals assigned to receive shock had lower systolic blood pressure (87.533 [±5.51]) before treatment, relative to control animals (106.412 [±2.80]; *F*_(1, 14)_ = 8.057 *p* = 0.0131). We adjusted for this effect, by computing a change from baseline score (Fig. 2B), which revealed a main effect of shock treatment (*F*_(1, 14)_ = 14.941, *p* = 0.0017). Blood flow did not differ before treatment (*F*_(1, 14)_ = 1.867, *p* > 0.05). Shock treatment also appeared to induce a rise in blood flow (Fig. 2D), an effect that approached statistical significance (*F*_(1, 14)_ = 4.129, *p* = 0.0616).

As previously observed for male animals, increased hemorrhage (as assessed by western blotting) was inversely related to the acute change in locomotor function observed 3 h after shock (*r* = −0.504, *p* = 0.0338) or capsaicin (*r* = −0.6254, *p* = 0.0168) treatment. Further, as observed in male animals, hemorrhage was tied to an increase in blood flow (*r* = 0.532, *p* = 0.0340). An increase in blood flow was also linked to the decrement in locomotor function in shocked animals (*r* = −0.504, *p* = 0.0467). In contrast, in capsaicin-treated animals, changes in cardiovascular function were not associated with hemorrhage or the degradation in locomotor performance (all absolute *r* values <0.356, *p* > 0.05).

Although the current study did not have sufficient *n* to conduct an exploratory mediational analysis,^41^ we were able to examine the relative fit of a model previously derived based on male animals. Treatment conditions for male rats (*N* = 64) mirrored the present experiments, with half the animals assigned to receive capsaicin or vehicle, whereas the remaining animals received shock or nothing. For both male and female animals, the mediation analysis was performed on the change in cardiac function (blood flow and systolic blood pressure) and locomotor performance (BBB score). The coefficients derived for female animals are given in bold (top) in Figure 2E, with the comparable values for male rats given directly below (gray).

The overall pattern for the derived values appears consistent across male and female animals, lending support to our previous analysis. What appears to differ is the relative contribution of the indirect (mediated) effect of hemorrhage, which seems to play a greater role in female animals. In addition, whereas hemorrhage is driven by pain-induced blood flow in both sexes, the effect of systolic blood pressure appears to vary with sex—in male animals a rise in systolic blood pressure directly impacted locomotor function independent of hemorrhage, whereas in female animals, the effect of a rise in systolic blood pressure on locomotor function was mediated by hemorrhage.

### Nociceptive stimulation impairs long-term recovery in female rats

In general, the acute effects of nociceptive stimulation mirrored those observed in male rats, producing an increase in the amount of hemorrhage observed at the site of injury and a decline in locomotor performance. In male rats, treatment with noxious electrical stimulation or capsaicin soon after injury also impairs long-term recovery.^2,7^ Experiments 3 and 4 examined the effects of capsaicin and shock, respectively, on recovery in female animals. Locomotor function was assessed daily with the BBB scale for the first week, again on day 10, and then once a week thereafter until day 28.

**FIG. 3.**
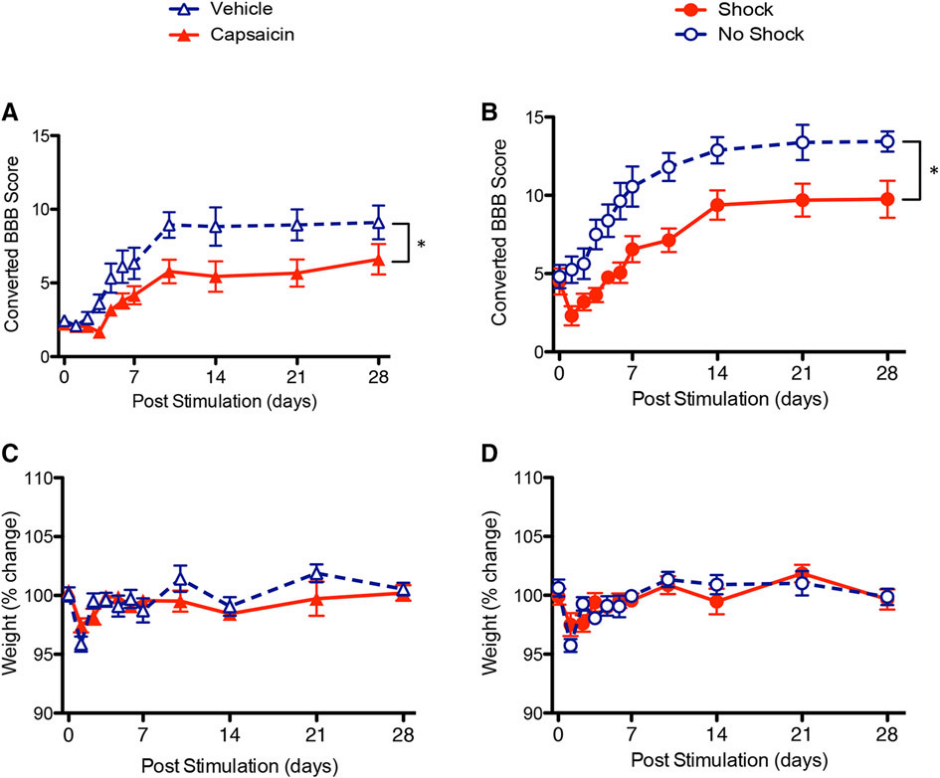
Effects of nociceptive stimulation on long-term recovery of locomotor function and weight. (**A**) Animals that were treated with capsaicin a day after injury exhibited poor recovery relative to their vehicle-treated controls. (**B**) Animals exposed to noxious electrical stimulation (Shock) a day after injury also exhibited poor recovery. (**C**) Recovery of weight after injury did not vary between capsaicin- and vehicle-treated animals. (**D**) Weight change did not differ between shocked and control animals. *Indicates statistical significance (*p* < 0.05). Error bars represent standard error of the mean (Capsaicin, *n* = 9; Vehicle, *n* = 9; Shock, *n* = 8; No Shock, *n* = 8). BBB, Basso, Beattie, Bresnahan.

Before capsaicin treatment in experiment 3, BBB scores ranged from 2.222 (±0.278) to 2.444 (±0.242) and did not differ between groups (*F*_(1, 16)_ < 1.0, *p* > 0.05). An ANCOVA using baseline BBB scores as the covariate revealed that animals injected with capsaicin recovered less function over time (*F*_(1, 15)_ = 5.764, *p* < 0.05; Fig. 3A). No other factors were significant (all *F*s < 1.525, *p* > 0.05).

Before shock exposure in experiment 4, baseline BBB cores ranged from 3.125 (±0.541) to 3.438 (±0.578). This group difference was not significant (*F*_(1, 14)_ < 1.0, *p* > 0.05). After stimulation, shocked animals recovered less locomotor function compared to controls (*F*_(1, 13)_ = 24.033, *p* < 0.0005; Fig. 3B).

Body weights were also measured throughout the recovery period. Previous research has shown that nociceptive stimulation slows recovery of weight in male rats.^2,7^ This effect was not observed in female animals. In both experiments 3 and 4, weights did not differ between groups before or after treatment (all *F*s < 4.543, *p* > 0.05; Figs. 3C,D).

### Estrous cycle modulates acute nociceptive-mediated deficits

Before injury, we monitored estrous cycle for 10 days. In experiment 1, animals were then randomly assigned to treatment conditions. Experiments 2–4 used a more sophisticated strategy, wherein animals were assigned to treatment conditions in a balanced manner, so that each of the four phases of the cycle (metestrus [M], diestrus [D], proestrus [P], and estrus [E]) were balanced across treatment conditions. This allowed us to explore whether our treatment effects vary with cycle.

**FIG. 4.**
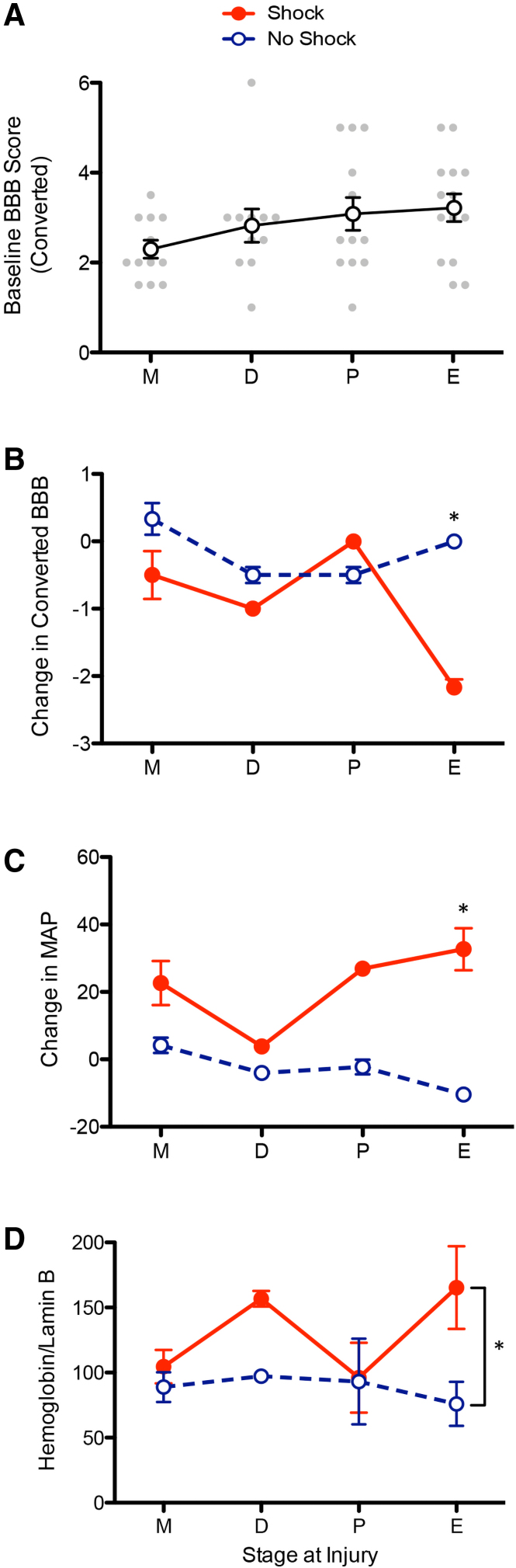
Effects of cycle on locomotor function and hemorrhage acutely post-SCI. (**A**) Before nociceptive input, baseline locomotor function varied according to stage of cycle at time of injury.^34,35^ (**B**) Exposure to noxious electrical stimulation (Shock) produced an acute disruption in locomotor performance, and this effect was most evident in animals that were injured in estrus. (**C**) Mean arterial pressure (MAP) also varied with stage of cycle, with a greater difference observed when animals were injured in estrus. (**D**) Whereas extent of hemorrhage varied some with cycle, only the main effect of shock treatment was statistically significant. *Indicates statistical significance (*p* < 0.05). Error bars represent standard error of the mean (Shock, *n* = 8; No Shock, *n* = 8). BBB, Basso, Beattie, Bresnahan; SCI, spinal cord injury.

We first examined whether the effect of injury on locomotor performance (24 h after injury) varied as a function of cycle (Fig. 4A). It appears that animals injured during metestrus (M), when both progesterone and estrogen are low,^42,43^ exhibited the greatest disruption in locomotor performance. Although the main effect of cycle was not statistically significant (*F*_(3, 46)_ = 1.637, *p* = 0.194), there was a significant linear trend (*F*_(1,46)_ = 4.603, *p* = 0.0372). Neither the quadratic nor the cubic trends were statistically significant (all *Fs* < 5.318, *p* > 0.05).

An additional analysis was conducted to determine whether the effect of shock treatment on acute locomotor performance varied as a function of cycle (Fig. 4B). The analysis revealed a significant Cycle × Shock Treatment interaction (*F*_(3, 8)_ = 10.917, *p* = 0.0034). This interaction emerged because shock treatment had a greater effect when administered during the estrus phase.

We then examined whether the effect of nociceptive input on cardiovascular function varied with cycle. We found that the effect of shock treatment on mean arterial blood pressure varied with cycle, yielding a significant Cycle × Shock Treatment interaction (*F*_(3, 8)_ = 4.575, *p* = 0.0380; Fig. 4C). Systolic blood pressure and flow also appeared to rise more in shocked animals injured in estrus, though these effects were not statistically significant (*F*_systolic (1, 8)_ = 4.5371, *p* = 0.0658; *F*_flow (1, 8)_ = 2.1022, *p* = 0.1851; data not shown).

An analysis of nociception-induced hemorrhage for animals treated with shock again suggested a modulatory effect of cycle at the time of injury. These trends were not, however, statistically significant (*F*s < 1.721, *p* > 0.05; Fig. 4D).

### Nociceptive input prolongs disruption of estrous cycle post-injury

**FIG. 5.**
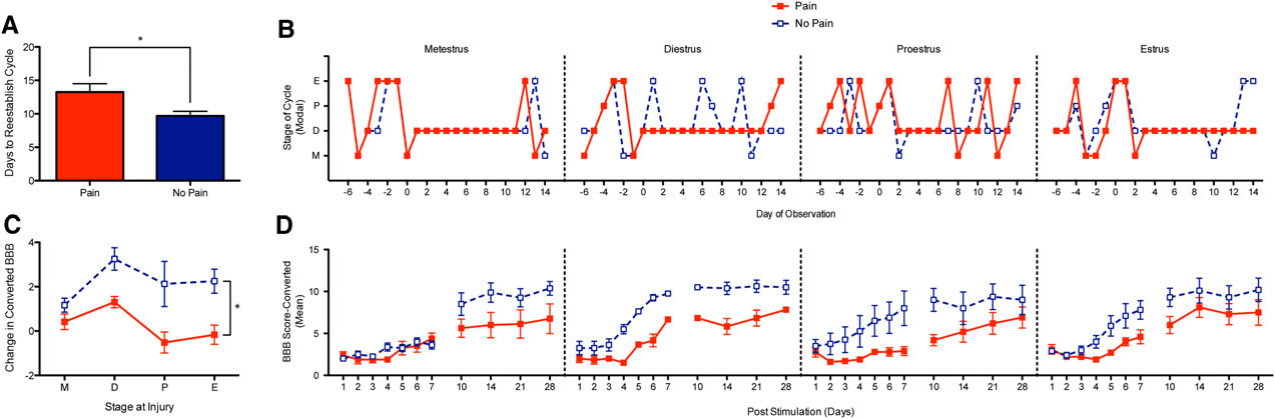
Interaction of nociceptive input with estrous cycle in female rats. (**A**) Post-injury, normal cycling is disrupted in animals for a period of ∼8–12 days. Resumption of normal cycling after injury-induced disruption took longer in animals that received noxious stimulation. (**B**) Disruption of normal estrous cycling shown across the different stages at time of injury. Injury occurred on day 0. (**C**) In the first 7 days of recovery, change in locomotor function differed according to cycle. Overall, animals that received nociceptive input recovered less function than animals exposed to noxious stimuli (shock or capsaicin). Animals that were injured during metestrus (M) exhibited poor performance over the subsequent week independent of whether they were exposed to stimulation a day after injury. (**D**) Interaction between stage of cycle at time of injury, nociceptive input, and locomotor recovery. Animals that received an injury in metestrus recovered function at a slower rate when they did not receive nociceptive input (No Pain). After the first week, comparable results were observed across cycle, with noxious stimulation having an adverse effect on long-term recovery. *Indicates statistical significance (*p* < 0.05). Error bars represent standard error of the mean (Capsaicin, *n* = 9; Vehicle, *n* = 9; Shock, *n* = 8; No Shock, *n* = 8). BBB, Basso, Beattie, Bresnahan.

Previous research has shown that the estrous cycle is disrupted after SCI. Injured animals exhibit either arrested cycling in diestrus or irregular cycling, with normal cycling resuming after 8–12 days.^23,24^ To determine whether nociceptive stimulation affected the recovery of cycling in experiments 3 and 4, the stage of the estrous cycle was assessed for at least 10 days before surgery and animals were balanced accordingly on the day of injury. Cycle was then tracked daily during recovery to assess the length of time before animals re-established normal cycling. We found that exposure to noxious stimulation increased the number of days until normal cycling returned (Figs. 5A,B). Because some animals did not begin to cycle until weeks after injury, the data had a positive skew. We addressed this issue by analyzing the log values. An ANOVA confirmed that animals that received nociceptive stimulation took longer to re-establish their estrous cycle (*F*_(1, 30)_ = 5.441, *p* < 0.05), and that this effect did not depend on noxious stimulation type (*F*_(1, 30)_ < 1.0, *p* > 0.05).

### Stage of cycle at injury impacts acute function, but not long-term recovery

We also examined whether estrous cycle at time of injury affected long-term recovery or the effect of nociceptive input. From the results obtained 24 h after injury (experiment 2), it appears that animals injured when estrogen and progesterone are low (metestrus) exhibit a greater loss in behavioral function. The same general pattern was evident when locomotor performance was assessed over a longer period (Fig. 5D), with animals injured in metestrus exhibiting delayed recovery during the first 7 days (mean performance during this period is shown in Fig. 5C). An ANCOVA, using baseline BBB as a covariate and day after injury as the repeated measure, revealed an interaction between stage and BBB score (*F*_(27, 225)_ = 1.642, *p* < 0.05). This interaction emerged because of variation in the rate of recovery during the first 7 days—animals that received a contusion injury while in metestrus, and that did not receive nociceptive input, showed less initial recovery of function.

From days 10 to 28, the magnitude of the group differences remained stable, demonstrating an effect of noxious exposure (*F*_(1, 26)_ = 10.444, *p* < 0.005), but not cycle (all *Fs <* 2.975, *p* > 0.05). Further analysis of the results obtained over the first week of recovery revealed a significant effect of day (*F*_(6, 156)_ = 66.06, *p* < 0.0001), and that the bulk of this effect was accounted for by the linear component (eta squared = 0.87). In addition, the linear component of the Nociception Type × Day, Cycle × Day, and the three-way interaction between Day, Nociception Type, and Cycle were all statistically significant (all *F*s > 6.95, *p* < 0.001). Given that pain administration prolonged the disruption of cycling, we also examined whether the effect of noxious stimulation on long-term recovery was better predicted by estrous cycle at the time of noxious input. When the data were recoded in this manner, we found that cycle accounted for just a fraction (27.9%) of the variance in long-term recovery.

## Discussion

In male animals, nociceptive input caudal to SCI increases tissue loss and impairs long-term recovery.^2^ These effects have been related to increased hemorrhage at the site of injury, potentially fueled by a rise in blood pressure/flow.^4,11^

### Nociceptive input negatively impacts female rats after spinal cord injury

Our first aim was to establish whether noxious input drives hemorrhage and undermines long-term recovery in female animals. To establish the generality of our results, we engaged nociceptive fibers after injury using either intermittent electrical stimulation or the irritant, capsaicin, applied 24 h after injury. Electrical stimulation allows precise control over temporal parameters and can be used to engage a broad range of fiber types in an intensity-dependent manner. In contrast, capsaicin selectively engages nociceptors that express the receptor transient receptor potential cation channel subfamily V member 1 receptor. Both treatments induced an acute disruption in locomotor performance and increased hemorrhage at the site of injury in female animals.

We also assessed blood pressure and flow for 3 h after treatment to explore whether locomotor deficits and hemorrhage were related to the effect of nociceptive stimulation on cardiovascular function. As observed in male animals,^11^ shock produced a rise in systolic blood pressure. Unlike male rats, we did not observe a significant change in blood flow. Statistical modeling confirmed that the overall effect of noxious input on hemorrhage and the acute disruption in locomotor function parallel the results obtained using male animals. In both cases, an increase in blood flow fostered hemorrhage independent of nociception, which, in turn, was inversely related to acute locomotor performance. What differed was the relative contribution of blood pressure versus blood flow—whereas a rise in systolic blood pressure plays a negligible role in driving hemorrhage in male animals, it plays a pivotal role in females. Further work is needed to discern the reason for this difference and whether it is related to relative size/weight.

Noxious stimulation also impairs long-term recovery in male animals, and the same effect is evident in females. Engaging nociceptive fibers with electrical stimulation or capsaicin a day after injury induced a lasting impairment in locomotor function, comparable in overall magnitude to that observed in male rats.^6,11,44^ Unlike male rats, there was no effect of nociceptive stimulation on body weight during the recovery period; independent of treatment, female rats stayed at approximately the same weight. In contrast, young adult (80–120 days of age) male rats typically gain weight, and this effect is stilted by exposure to noxious stimulation after injury.

In past studies, we have examined the cellular processes engaged by noxious stimulation in contused animals, guided by earlier work demonstrating that both noxious electrical stimulation and application of the irritant, capsaicin, induce a form of neural excitation within the dorsal horn of the spinal cord.^3,45–47^ We have suggested that this state of overexcitation can foster cell death and induce a state of maladaptive plasticity that fosters the development of chronic pain after SCI.^3,45^ Supporting this, we have shown that noxious stimulation engages signal pathways indicative of apoptosis and pyroptosis.^1,7^ Stimulation also engages signal pathways indicative of nociceptive sensitization and the expression of proinflammatory cytokines (e.g., tumor necrosis factor [TNF], interleukin).^1,48^ Interestingly, noxious stimulation applied to the tail or hind leg appears to have a diffuse effect, inducing the expression of TNF in both the lumbosacral spinal cord and rostral contused tissue. Additional studies have linked these effects to increased expression of Ca^++^-permeable aminomethylphosphonic acid receptors within the ventral horn.^49^

Other recent studies have explored how noxious stimulation engages a rise in systolic blood pressure and the contribution of these effects to hemorrhage and decline in locomotor function.^11,50^ We hypothesized that the hemodynamic response may be mediated by efferent projections caudal to T2, producing an unregulated rise in heart rate and blood pressure akin to autonomic dysreflexia.^51^ If this was the case, noxious stimulation should induce these effects in T2 transected animals. To our surprise, both the hemodynamic response and hemorrhage were blocked when communication with the brain was blocked by a surgical transection.^40,50^ Similar results were obtained when a pharmacological transection was performed by slowly infusing the anesthetic, lidocaine, onto the spinal cord tissue at T2.^44^ On the basis of these findings, we have suggested that the adverse effect of nociceptive input after injury depends, in part, on brain systems. Interestingly, these brain-dependent effects do not appear to depend upon psychological pain/affect, because an analgesic dose of morphine does not block hemorrhage or the adverse effect nociceptive stimulation has on recovery.^4,52^ Further work is needed to uncover whether brain systems fuel tissue loss through descending fibers and/or the engagement of systemic processes.

### Sex as a biological variable

Overall, noxious stimulation had comparable effects on acute hemorrhage, cardiac function, and long-term recovery in male and female animals. This general pattern of results fits with other work demonstrating similar recovery after SCI. After a moderate contusion at T10, a comparison of age-matched male and female rats found no difference in recovery of locomotor function, nor in lesion area, after histological analysis.^53^ Likewise, administration of estradiol or progesterone in male and ovariectomized female rats produced no observable long-term advantages in functional recovery.^54,55^ Further, in mice, no differences in functional recovery or histological analysis were observed between male and female contused animals.^56^ The fact that nociceptive input has an adverse effect after injury in both male and female animals highlights the need to attend to this factor regardless of sex—to reduce afferent drive and minimize exposure to circumstances that may engage nociceptive activity and/or stress after injury.

Although we and others observe few sex differences in the overall response to injury or noxious stimulation, the variable nature of gonadal hormones and their effect on pain reactivity may explain why some sex-dependent effects were obscured in our initial analyses. A more detailed analysis revealed that the effect of injury and noxious stimulation on female animals varied as a function of estrous cycle. Animals injured in metestrus exhibited a greater disruption in locomotor performance 24 h later, and this effect persisted throughout the first week of recovery. We also observed that the acute effects of nociceptive input on locomotor function, blood pressure, and hemorrhage were generally greater in animals injured in estrus. This is consistent with evidence found in the pain literature, where female animals exhibit greater hypersensitivity after exposure to noxious stimulation.^26–29^ These studies suggest that when sex hormones are rapidly changing (i.e., proestrus) or present in lower levels (i.e., estrus), the animal is more susceptible to the effects of pain.

A limitation of the current experiment stems from the original aim—to determine whether noxious stimulation induces hemorrhage and impairs recovery in female animals. Although we balanced the assignment of animals to treatment condition, we were not expecting to observe an effect of estrous cycle. Consequently, we did not take blood samples to assess hormone levels. Further, although the nature of the design yielded reasonable sample sizes for assessing the effect of estrous cycle on the acute (Fig. 4A) and long term (Fig. 5C,D) effect of injury, and the effect of nociceptive input on estrous cycle (Fig. 5A,B), our analyses of the effect of shock treatment (Fig. 4B–D) were potentially underpowered. Although we gained some statistical power using trend analyses, further work is needed to verify that the acute effect of noxious stimulation after SCI varies across the estrous cycle in female animals.

Interestingly, estrous cycle and nociceptive input appeared to interact in a bidirectional manner; whereas estrous cycle modulated the effect of nociceptive stimulation on injury, nociceptive input influenced the recovery of normal cycling. This disruption in estrous cycle is consistent with past work demonstrating a delay in normal cycling after SCI and a disruptive effect of stress.^12,14,15,57,58^ In both cases, arrested cycling is typically held in a diestrus-like state,^23,58^ in part attributable to disrupted innervation to the ovaries.^25^ Recovery of regular cycling often occurs along with recovery of hindlimb function or within a period of ∼8–12 days.^23^ In studies that found improved recovery in females, sex-dependent differences in locomotor recovery were typically evident after 2 weeks.^59^ Noxious stimulation caused both impairment in recovery that continued throughout the 28-day study as well as a delay in the resumption of normal estrous cycling. These results suggest that nociception compounds the impact of SCI on estrous, suppressing the system's capability to re-establish normal hormonal levels that might contribute some benefit to recovery.

## Conclusion

Subsequent to the 2015 NIH call for researchers to examine sex as a biological variable, studies of neurological conditions have reported sex-dependent differences with important implications for clinical translation.^60^ The body of work surrounding sex-dependent differences post-trauma to the spinal cord yields conflicting evidence. Our overall results are consistent with other reports demonstrating comparable findings after SCI in both male and female animals.^53–56^ Yet, this conclusion obscures modulatory effects linked to the cycling of female hormones; when collapsed across cycle, male and female animals may not differ. A more detailed analysis reveals an effect of estrous cycle in female animals. Further work is needed to detail how cycle-dependent processes influence injury, to potentially augment or diminish tissue loss and long-term recovery.

## Abbreviations Used

ANOVA: analysis of variance
ANCOVA: analysis of covariance
BBB: Basso, Beattie, Bresnahan
i.p.: intraperitoneal
MASCIS: Multicenter Animal Spinal Cord Injury Study
NIH: National Institutes of Health
SCI: spinal cord injury
TBS: Tris-buffered saline
TNF: tumor necrosis factor
